# The complete chloroplast genome of a karst-dwelling plant *Primulina tenuituba* (Gesneriaceae)

**DOI:** 10.1080/23802359.2021.2011446

**Published:** 2022-03-17

**Authors:** Tao Peng, Luyan Tang, Zhiruo Zeng, Tianyun Zhang, Jinyi Wang, Xuping Zhou, Bin Zhu, Xueye Du, Fang Wen

**Affiliations:** a School of Life Sciences, Guizhou Normal University, Guiyang, Guizhou, China; b Biodiversity Research Center, Guizhou Normal University, Guiyang, China; c Guangxi Key Laboratory of Plant Conservation and Restoration Ecology in Karst Terrain, Guangxi Institute of Botany, Guangxi Zhuang Autonomous Region and Chinese Academy of Sciences, Guilin, China; d Gesneriad Committee of China Wild Plant Conservation Association, National Gesneriaceae Germplasm Resources Bank of GXIB, Gesneriad Conservation Center of China (GCCC), Guilin Botanical Garden, Guangxi Zhuang Autonomous Region and Chinese Academy of Sciences, Guilin, China

**Keywords:** *Primulina tenuituba*, chloroplast genome, phylogenetics

## Abstract

*Primulina tenuituba* is a species in the Gesneriaceae family that is widely distributed in China. It is a karst-dwelling species with an enormous tolerance for extreme drought and high temperatures. The species is also used in traditional Chinese medicine. In this study, the complete chloroplast genome of *P. tenuituba* was assembled and characterized for the first time. The complete chloroplast genome exhibited a typical quadripartite cycle of 153,236 bp in length, including a pair of inverted repeats (IRs) of 25,494 bp, which were separated by a large single-copy (LSC) region of 84,364 bp and a small single copy (SSC) region of 17,884 bp. The GC content was 37.6%. The complete chloroplast genome of *P. tenuituba* contains 114 genes, including 80 protein-coding genes, 30 tRNAs genes, and four rRNAs. The phylogenetic analysis showed that *P. tenuituba* is closely related to *P. eburnea*. The newly reported chloroplast genome of *P. tenuituba* would provide valuable data for further studies on its evolution and adaptation mechanism.

*Primulina tenuituba* (W.T. Wang) Yin Z. Wang (Gesneriaceae), a karst-dwelling species, is widely distributed in Guizhou, Hunan, Sichuan, and Hubei Provinces in China, and appears to have a strong tolerance for extreme drought and high temperatures. Its rhizome is also used in traditional Chinese medicine in treating rheumatism (Editorial Committee of the Flora of China 1990; Li and Wang 2004). However, its phylogenetic relationship in the Gesneriaceae family is unclear due to the absence of genome (plastome) information. After our research, we can now report the complete chloroplast genome of *P. tenuituba* to clarify its phylogenetic placement in Gesneriaceae. The plants were collected from Guiyang, Guizhou, China (26°35′4.43″ N, 106°43′35.20″ E), and a voucher specimen (WF200808-12) was deposited at the herbarium of Guizhou Normal University (GNUB, https://sjxy.gznu.edu.cn/, and emailed to contact person Tao Peng, pengtao@gznu.edu.cn). Total genomic DNA was extracted from the fresh leaves of *P. tenuituba* using a modified cetyltrimethylammonium bromide (CTAB) method (Doyle and Doyle 1987) and sent to Majorbio Company (http://www.majorbio.com/, China) for next-generation sequencing. Short-insert (350 bp) paired-end read libraries preparation and 2 × 150 bp sequencing were performed on an Illumina (HiSeq4000) genome analyzer platform. A total of 13,101,144 raw reads were obtained, and 98.27% of which were declared as clean reads. The clean reads were mapped to *P. ophiopogoides* (D. Fang & W.T. Wang) Yin Z. Wang (MF227819, complete chloroplast genome reference) in Geneious Primer (Kearse et al. 2012) to exclude nuclear and mitochondrial reads. Then *de novo* was performed for assembling construction. Contigs were concatenated using the Repeat Finder option implemented in Geneious Primer until only one contig (including SSC, IR, and LSC) was built. The IR region was detected by the Repeat Finder option in Geneious Primer and was reverse copied to obtain the complete plastid genome. The annotation approach was performed using CPGAVAS2 and PGA (Qu et al. 2019; Shi et al. 2019).

The complete chloroplast genome of *P. tenuituba* was 153,236 bp in length (GenBank-MW245830), containing one large single-copy (LSC) region of 84,364 bp and one small single-copy (SSC) region of 17,884 bp, which are separated by two inverted repeat (IR) regions of 25,494 bp. The GC content was 37.6%. A total of 114 unique genes were annotated in the complete chloroplast genome, including 80 protein-coding genes, four ribosomal RNA genes, and 30 transfer RNA genes.

To investigate the phylogenetic relationships with the closely related species, the maximum likelihood (ML) phylogenetic tree was constructed by RAxML (Stamatakis 2006) based on 13 complete chloroplast genomes in Gesneriaceae (Figure 1). *Aphelandra Knappiae* Wassh. representing Acanthaceae and *Premna microphylla* Turcz. and *Stachys sylvatica* L. representing Labiatae were employed as outgroups. The result showed that Gesneriaceae is monophyletic. *Primulina tenuituba* fell into a monophyletic group *Primulina* and was closely related to *P. eburnea* (Hance) Yin Z. Wang. This relationship was congruent with their morphological similarity, such as the narrowly long leaves. The newly reported complete chloroplast genome of *P. tenuituba* would provide valuable data for further studies on its evolution and adaptation mechanism.

**Figure 1.**
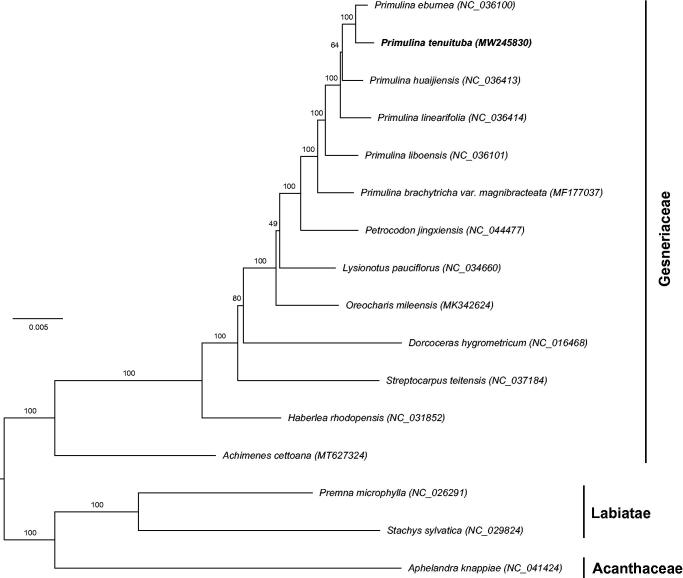
Phylogenetic tree reconstructed by maximum likelihood (ML) analysis based on the dataset of whole-chloroplast protein-coding genes from 13 species of Gesneriaceae, two species from Labiatae and one of Acanthaceae, numbers upon branches are assessed by ML bootstrap.

## Data Availability

The genome sequence data supporting this study’s findings are openly available in GenBank of NCBI at (https://www.ncbi.nlm.nih.gov/) under the accession no. MW245830. The associated BioProject, SRA, and Bio-Sample numbers are PRJNA682283, SRR13188905, and SAMN16986174, respectively.
